# A Phase Ib Investigator-Initiated Trial of Filgotinib in Patients With Idiopathic Multicentric Castleman Disease

**DOI:** 10.7759/cureus.78865

**Published:** 2025-02-11

**Authors:** Shoichi Fukui, Remi Sumiyoshi, Tomohiro Koga, Naoki Hosogaya, Sawana Narita, Shimpei Morimoto, Hiroshi Yano, Atsushi Katsube, Shingo Yano, Yasufumi Masaki, Shinichiro Tsunoda, Shuzo Sato, Kiyoshi Migita, Kazuko Matsuyama, Naoki Kato, Atsuhiko Kawamoto, Atsushi Kawakami

**Affiliations:** 1 Department of Immunology and Rheumatology, Nagasaki University Graduate School of Biomedical Sciences, Nagasaki, JPN; 2 Clinical Research Center, Nagasaki University Hospital, Nagasaki, JPN; 3 Divison of Clinical Oncology/Hematology, Department of Internal Medicine, The Jikei University School of Medicine, Tokyo, JPN; 4 Department of Hematology and Immunology, Kanazawa Medical University, Uchinada, JPN; 5 Divison of Clinical Immunology and Hematology, Department of Internal Medicine, Sumitomo Hospital, Osaka, JPN; 6 Department of Rheumatology, Fukushima Medical University School of Medicine, Fukushima, JPN; 7 Translational Research Center for Medical Innovation, Foundation for Biomedical Research and Innovation at Kobe, Kobe, JPN

**Keywords:** albumin, c-reactive protein, hemoglobin, janus kinase, performance status

## Abstract

Background

Idiopathic multicentric Castleman disease (iMCD) is a chronic systemic inflammatory disease characterized by the production of interleukin (IL)-6. The contribution of Janus kinase, downstream of IL-6 signaling, to the pathophysiology of iMCD has been suggested by several studies.

Patients and methods

This phase Ib single-arm trial evaluated the safety profile and efficacy of filgotinib, a JAK1 preferential inhibitor, in patients with iMCD. We recruited patients with disease activity evaluated based on their values of C-reactive protein (CRP), hemoglobin, albumin, and Eastern Cooperative Oncology Group performance status (ECOG-PS). Filgotinib (200 mg daily) was administered for eight weeks.

Results

Five patients who were newly diagnosed with or under treatment for iMCD were recruited. The lymph node histology of all five patients was the plasma-cell type. Filgotinib demonstrated a favorable safety profile with manageable adverse events. At eight weeks, improvements in the ECOG-PS were observed in two patients, but no improvements in CRP, hemoglobin, or albumin levels were observed.

Conclusion

The safety profile of filgotinib against iMCD was comparable to those against rheumatoid arthritis and ulcerative colitis over a short duration, but the efficacy of filgotinib against iMCD was not evident after eight weeks. Long-term evaluations of the safety profile and efficacy of filgotinib in the treatment of iMCD are necessary.

## Introduction

Castleman disease, first described in 1956 [1], is a chronic inflammatory disease associated with lymphadenopathy. The clinical form of Castleman disease is divided into unicentric and multicentric Castleman disease, and multicentric Castleman disease is further divided into human herpesvirus (HHV)-8-associated multicentric Castleman disease and idiopathic multicentric Castleman disease (iMCD) [2]. iMCD manifests as nonspecific symptoms suggestive of an inflammatory disease, such as fever, night sweats, weight loss, and malaise. It is a rare and refractory disease that annually affects ~900-4200 people in the US [3] and ~1500 in Japan [4]. If not treated appropriately, iMCD may lead to organ damage and secondary amyloidosis, thereby reducing patients' quality of life (QOL) and shortening their life expectancy.

An increase in interleukin (IL)-6 levels in patients with Castleman disease has been reported [5], and a correlation between IL-6 levels and clinical symptoms has also been reported [6]. Based on these findings, the IL-6-targeting agents tocilizumab [7] and siltuximab [8] (siltuximab is not approved for use in Japan) were developed for patients with iMCD. IL-6 inhibitors are effective drugs but decreases in the diameter of lymph nodes to <10 mm were observed in 52.2% of patients in a tocilizumab trial [7], and ﻿durable tumor and symptomatic responses occurred in 34% of the patients in a siltuximab trial [9].

For refractory iMCD, chemotherapy that has been used to treat malignant lymphoma and multiple myeloma (cyclophosphamide, doxorubicin, vincristine, etoposide, rituximab, bortezomib, thalidomide, etc.) has been attempted [10], but the evidence regarding these treatments is minimal, and no treatment strategy for refractory iMCD has been established. There is an urgent need to develop an effective treatment for iMCD.

Janus kinases (JAKs) are downstream regulators of multiple cytokine receptors, including IL-6 receptors [11]. The interaction of cytokine receptors with their ligands results in the activation of receptor-associated JAKs and tyrosine phosphorylation, which in turn activates signal transcription factors [12]. Pierson et al.'s study suggested that JAKs contribute to the activation of signal transducer and activator of transcription (STAT) 3 in the pathogenesis of iMCD [13], and that the inhibition of JAK-STAT signaling represents a promising approach for treating iMCD. Of the four subtypes of the JAK family (JAK1, JAK2, JAK3, and TYK2), JAK1 is involved in signaling through inflammatory cytokines such as IL-6, and it is known to cause lymphocyte activation and proliferation in rheumatoid arthritis (RA).

JAK inhibitors have emerged as new oral therapeutic drugs for RA [14]. Filgotinib is a JAK inhibitor that is more preferential for JAK1 than other JAK inhibitors [15]. JAK2 is required for erythropoiesis, myelopoiesis, and platelet production, and JAK3 is important for lymphocyte proliferation and homeostasis [16]; inhibition of the JAK2 and JAK3 pathways can cause safety issues [17]. JAK1 preference is considered an essential property for the safe treatment of iMCD, particularly since anemia (62%-87%) and thrombocytopenia (22%-44%) often develop in patients with iMCD [18]. Based on this rationale and the above-described findings, we conducted a phase Ib investigator-initiated trial of filgotinib treatment for iMCD.

## Materials and methods

Ethics

The trial was performed in accord with the principles of the Declaration of Helsinki and the Good Clinical Practice guidelines. The ethics committees and institutional review boards at the participating sites approved the research protocol (approved by Nagasaki University Hospital Institutional Review Board, approval no. 123-002). Before entry, written informed consent was obtained from all patients.

Trial design and patients

This prospective, single-arm trial was conducted at Nagasaki University Hospital, The Jikei University Hospital, Kanazawa Medical University Hospital, Sumitomo Hospital, and Fukushima Medical University Hospital. This trial was registered in the Japan Registry of Clinical Trials as jRCT2071230108. All patients provided signed informed consent prior to their enrollment. Filgotinib (200 mg once daily) was given for eight weeks. The screening period for trial eligibility did not exceed 14 days. The Translational Research Center for Medical Innovation (TRI, Hyogo, Japan) conducted the trial with guidance from the Clinical Research Center of Nagasaki University Hospital.

Patients who meet all of the following criteria were recruited: (1) patients newly diagnosed or under treatment for iMCD according to the diagnostic criteria for idiopathic multicentric Castleman disease issued by Japan's Ministry of Health, Labour and Welfare, i.e., "Designated intractable diseases that became effective on April 1, 2018 (notice no. 331)"; (2) patients aged ≥18 years and <65 years at the time of informed consent; (3) patients with a total score on the C-reactive protein, hemoglobin, albumin, + ECOG-PS (Eastern Cooperative Oncology Group-Performance Status) (CHAP) [4] that was ≥2 points in total with hemoglobin or albumin ≥1 point, and CRP ≥1 point at the screening; (4) patients with a negative pregnancy test (females of childbearing potential) and their agreement to use adequate contraception (both females of childbearing potential and males) during the study and for 30 days after they had taken their last dose of filgotinib.

Patients with any one of the following items were excluded: known hypersensitivity to filgotinib, prior exposure to filgotinib, insufficient washout period for other drugs (tocilizumab and other immunosuppressants), other autoimmune diseases than iMCD, uncontrolled infectious diseases, the inappropriate function of an organ or bone marrow (kidney, liver, neutropenia, lymphopenia, and anemia), definite iMCD-thrombocytopenia, anasarca, fever, reticulin fibrosis, renal insufficiency, and organomegaly (TAFRO) clinical subtype defined by the validated international definition [19], a recent vaccination with a live vaccine, participation in another clinical study, or postpartum and lactating patients (Table 1).

**Table 1:** Exclusion criteria eGFR - estimated glomerular infiltration rate; iMCD - idiopathic multicentric Castleman disease; JAK - Janus kinase; TAFRO - the thrombocytopenia, anasarca, fever, reticulin fibrosis, renal insufficiency, and organomegaly clinical subtype; TNF-α - tumor necrosis factor-alpha.

Exclusion criteria
1. Known hypersensitivity to filgotinib or a history of severe allergic reaction to any drug
2. Prior exposure to filgotinib
3. Patients who had received tocilizumab within 28 days before enrollment
4. Patients with a history of malignancy within the last five years (with the exception of patients with non-melanoma skin cancer or carcinoma in situ of the cervix that has responded to treatment)
5. History of lymphoproliferative disease other than iMCD
6. Patients with autoimmune disease other than iMCD (rheumatoid arthritis, systemic lupus erythematosus, Sjogren's syndrome, dermatomyositis/polymyositis, vasculitis, IgG4-related disease, Behcet's disease, etc., but not limited to these)
7. Patients positive for HBs antigen (patients negative for HBs antigen and positive for anti-HBs antibody and/or anti-HBc antibody could participate in this study.)
8. Patients with uncontrolled infectious diseases (tuberculosis, human immunodeficiency virus, etc., but not limited to these)
9. Patients with any of the following laboratory abnormalities at screening: eGFR<60 mL/min/1.73 m^2^, moderate or severe hepatic impairment (≥Child-Pugh class B), neutrophil count <1,000/mm^3^, lymphocyte count <500/mm^3^, Hemoglobin <8.0 g/dL
10. Definite iMCD-TAFRO defined by "validated international definition of the thrombocytopenia, anasarca, fever, reticulin fibrosis, renal insufficiency, and organomegaly clinical subtype of idiopathic multicentric Castleman disease"
11. Patients who had newly started glucocorticoids within 14 days before enrollment (however, topical preparations and use of continuous glucocorticoids for the treatment of iMCD were allowed)
12. Patients who had received immunosuppressants such as rituximab, sarilumab, or cyclophosphamide, TNF-α inhibitors such as infliximab, etanercept, adalimumab, golimumab, certolizumab, ozoralizumab, vedolizumab, or abatacept within 28 days before enrollment,
13. Patients who had received an immunosuppressant (tacrolimus, cyclosporine, azathioprine, mizoribine, or sirolimus), JAK inhibitor (tofacitinib, baricitinib, peficitinib, upadacitinib, ruxolitinib, abrocitinib, ritrecitinib, or delgocitinib), or carotegrast within 14 days before enrollment (topical use of immunosuppressants was permitted.)
14. Patients who had received a live vaccine within seven days before enrollment and patients who were scheduled to receive a live vaccine during the period from the enrollment date to the end date of the study
15. Patients who had participated in another clinical study or clinical research within 8 weeks before enrollment
16. Patients who were >6 months postpartum at the time of enrollment, patients who had had an abortion within 6 months before enrollment, and female patients who had been lactating or pregnant
17. Other patients considered ineligible for the study by the investigator or sub-investigator

Endpoints

Adverse events (the proportions of adverse events, serious adverse events, adverse drug reactions, adverse events leading to discontinuation of the investigational product, and adverse events leading to death) and abnormalities in laboratory test results (complete blood count, blood biochemistry tests, contrast-enhanced computed tomography, 12-lead ECG, and qualitative urinalysis) as the study's primary endpoint. The secondary endpoints were the following changes that occurred between the baseline and day 56 of treatment: (1) the patient's CHAP score, (2) C-reactive protein (CRP; mg/dL), (3) hemoglobin (g/dL), (4) albumin (g/dL), (5) the ECOG-PS value, (6) the Short Form 36-item health survey (SF-36) [20], (7) the Physician's Global Assessment (a 100-mm visual analog scale (VAS)), (8) the Patient's Global Assessment (a 100-mm VAS), and (9) the proportion of patients achieving a one-point reduction in their CHAP score at week eight compared to the baseline value.

Discontinuation criteria

The criteria used for patient discontinuation were: (1) the patient withdrew consent, (2) a life-threatening adverse event requiring emergency treatment occurred, (3) the patient had a 'rest' period of >2 consecutive weeks of filgotinib or missed doses at the patient's intention, (4) the observation of a serious or medically significant adverse event as judged by an investigator or sub-investigator to be causally related to the filgotinib that did not recover to a moderate or less severe level within two weeks after withdrawal of the filgotinib, (5) the use of prohibited concomitant drugs or the initiation or a dose increase of oral glucocorticoids was required due to worsening of the primary disease, (6) the patient became pregnant while participating in the study, (7) the patient was found to have an uncontrolled infectious disease during the study participation period, and (8) an investigator or sub-investigator judged that it was difficult or inappropriate for the patient to continue the study for other reasons.

Statistical analysis

Categorical variables are described as frequencies and proportions of the number of patients analyzed, and continuous variables are presented as medians with minimum and maximum values. Data analyses were performed using SAS version 9.4 (SAS Institute Inc., Cary, North Carolina).

## Results

Patient characteristics and baseline status

The trial was conducted from December 22, 2023, until June 21, 2024 (the last trial visit). Six patients were screened for eligibility for inclusion in this study. One patient was not eligible because of anemia. The final study population was thus five patients with iMCD. Table 2 presents the results of the baseline measurements. The median patient age was 60 years. Two of the patients were treated with prednisolone. Three patients were treatment-naïve. The lymph node histology at diagnosis was the plasma-cell type, and neither the hyaline-vascular type nor the mixed type was included. The median CRP level was 6.73 mg/dL. The median CHAP score at the baseline was five points. One patient was concomitantly treated with filgotinib and 10-mg prednisolone daily.

**Table 2:** Baseline characteristics of patients with idiopathic multicentric Castleman disease treated with filgotinib (n=5) CRP - C-reactive protein; ECOG-PS: Eastern Cooperative Oncology Group-Performance Status; CHAP - CRP, hemoglobin, albumin, + ECOG-PS; VAS - visual analog scale; SF-36 - Short Form 36-item health survey

Characteristic	Median (min−max) or n (%)
Age, median (min−max)	60 (37–61)
Sex, female, n (%)	2 (40%)
Height, cm, median (min−max)	169.8 (151.7–175.6)
Body weight, kg, median (min−max)	73.7 (50.9–75.0)
Body mass index, median (min−max)	24.0 (19.6–27.4)
Treatment-naïve, n (%)	3 (60%)
Previous treatment with immunosuppressant, n (%)	
Prednisolone	2 (40%)
Others	0 (0%)
Concomitant use of immunosuppressant, n (%)	
Prednisolone 10 mg/day	1 (20%)
Others	0 (0%)
Histology, n (%)	
Hyaline vascular type	0 (0%)
Plasma cell type	5 (100%)
Mixed type	0 (0%)
CRP, mg/dL, median (min−max)	6.73 (2.47–7.45)
Hemoglobin, g/dL, median (min−max)	10.1 (8.1–11.8)
Albumin, g/dL, median (min−max)	2.9 (2.5–3.3)
ECOG-PS	
0	2 (40%)
1	3 (60%)
CHAP score, median (min−max)	5 (3–6)
Patient VAS, mm, median (min−max)	30 (11–58)
Physician VAS, mm, median (min−max)	6 (4–33)
SF-36 Physical component summary score, median (min−max)	43.7 (38.5–57.6)
SF-36 Mental component summary score, median (min−max)	47.8 (32.2–61.8)
SF-36 Role/Social component summary score, median (min−max)	54.2 (51.9–57.0)

Retention rate

All five patients completed the eight weeks (56 days) of filgotinib treatment, with no discontinuation.

Safety

All five patients experienced adverse events, and four patients had adverse drug reactions (Table 3). Of the nine adverse events, six were recognized as adverse drug reactions. One patient experienced a herpes zoster infection, who was not treated with concomitant prednisolone and had no other risk factors for infectious diseases. All adverse events were mild. In the laboratory tests, one patient showed a low red blood cell count, and three showed low hemoglobin and hematocrit levels. However, these adverse events were not recognized as adverse drug reactions. All five patients showed a normal white blood cell count, platelet count, sodium, potassium, chloride, blood urea nitrogen, creatinine, total bilirubin, aspartate aminotransferase, alanine aminotransferase, lactate dehydrogenase, alkaline phosphatase, γ-glutamyl transpeptidase, and creatine kinase for eight weeks. In the urinalyses, the results of protein were '−', '± ', or '1+' for eight weeks in all five patients.

**Table 3:** Adverse events and adverse drug reaction (n=5) The data are n, %. The terminology is based on the Medical Dictionary for Regulatory Activities/Japanese version LLT (MedDRA/J) *adverse drug reaction assessed as causally related to filgotinib.

Adverse event	n (%)
Headache*	2 (40%)
Dizziness*	1 (20%)
Pyrexia*	1 (20%)
Herpes zoster*	1 (20%)
Gastralgia*	1 (20%)
Pharyngodynia	1 (20%)
Sleepiness	1 (20%)
Common cold	1 (20%)

Treatment response

**Figure 1:**
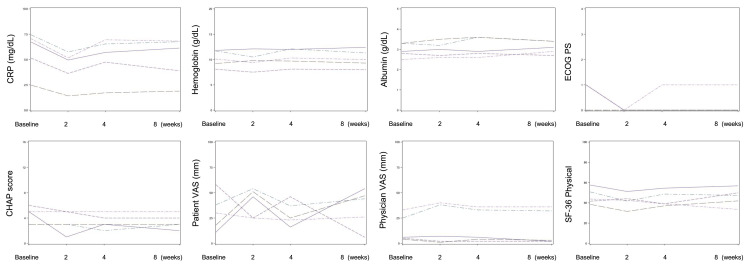
The time series of outcome measures in this trial of filgotinib against idiopathic multicentric Castleman disease (iMCD) CRP - C-reactive protein; ECOG-PS: Eastern Cooperative Oncology Group-Performance Status; CHAP - CRP, hemoglobin, albumin, + ECOG-PS; VAS - visual analog scale; SF-36 - Short Form 36-item health survey

Figure 1 illustrates the time series of the clinical parameters at baseline and at two, four, and eight weeks of treatment with filgotinib. All patients had high CRP levels at baseline, and no apparent reduction in CRP levels at eight weeks was observed. The patients' hemoglobin and albumin levels were unchanged at eight weeks versus those at baseline. Two patients showed a reduction in the ECOG PS from two to one. One point of decrease in the CHAP score was observed in two patients. No meaningful changes were observed in the patient VAS, physician VAS, or SF-36.

## Discussion

This trial is the first prospective study to evaluate the safety profile and efficacy of the JAK1 preferential inhibitor filgotinib for the treatment of iMCD. Although a herpes zoster infection occurred as an adverse drug reaction in one case, all five patients tolerated the filgotinib. Regarding the treatment response, except for reductions in the ECOG-PS in two patients at eight weeks, the CRP, hemoglobin, albumin, patient VAS, physician VAS, and SF-36 values were comparable at eight weeks compared with those obtained at baseline.

The profile of adverse events in this trial was consistent with previous reports concerning RA [14] and ulcerative colitis (UC) [21]. The rate of herpes zoster infection was 0.7%-1.3% at 12 weeks of treatment in a trial of filgotinib for RA [14] and 0.2%-0.6% at 10.8 weeks of filgotinib treatment in an investigation of UC [21]. A single case of herpes zoster was observed in the present patient series. Vaccination against herpes zoster would be necessary in this patient population, as was suggested in patients with RA treated with JAK inhibitors [22]. Concerning other adverse events, two headaches occurred among our five patients; headache is also a common adverse event associated with filgotinib [14]. In our comparison of the adverse events observed in this trial with those in previous trials, we did not identify any unexpected adverse events. However, further studies with larger sample sizes and longer durations are required to elucidate the safety profile of filgotinib in the treatment of iMCD.

We did not observe any apparent improvement in clinical conditions after the eight-week filgotinib treatment for iMCD in this trial. Although the reductions in the ECOG-PS in two patients led to a reduction of one point in the CHAP score at eight weeks (the ECOG-PS is one of the components of the CHAP score), no evident improvement in the CRP, hemoglobin, or albumin levels was observed in any of the patients. Given the results of trials of tocilizumab [7] and siltuximab [9] for iMCD that demonstrated remarkable improvements in CRP, hemoglobin, and albumin levels, such an effect of filgotinib against iMCD was not evident.

In addition, considering the report that the disease activity evaluated based on the Disease Activity Score in 28 joints CRP (DAS28-CRP) improved continuously from four weeks to 12 weeks in patients with RA [14], we regard the change in CRP in this trial as an indicator of a lack of response to filgotinib. Regardless of the two patients' improvement in the ECOG-PS, both the patient and physician VAS and the SF-36 measurements showed no changes in the same patients at eight weeks. This inconsistency among the parameters' results also makes it difficult to interpret the effects of filgotinib on iMCD.

Two speculations may explain the patients' insufficient response to filgotinib in this trial. First, the relative importance of JAK2, rather than JAK1, was suggested. The successful treatment of iMCD-TAFRO with ruxolitinib (a potent and preferential JAK1/2 inhibitor approved for the treatment of myelofibrosis [23]) has been reported [24,25]. The effectiveness of ruxolitinib in a patient with iMCD-not otherwise specified (NOS) was also described [26]. In light of these case reports, filgotinib's property of being preferential for JAK1 might have been insufficient to inhibit JAK2 in iMCD in a hypothesis. Second, an insufficient filgotinib dose may have been used in this trial. A dose-dependent improvement of outcomes was suggested in a trial of filgotinib for RA [14]. Furthermore, the approved dose of intravenous tocilizumab for iMCD is once every two weeks or every week if necessary; however, that for RA is once every four weeks. Given the differences in pathophysiology between iMCD and RA, a dose >200 mg/day might have been necessary for iMCD.

This study has several limitations. Although one of the purposes of this trial was the development of a new medication for patients with iMCD that is refractory to tocilizumab, all five of the recruited patients were naïve to tocilizumab. Second, according to trials concerning RA and UC, which evaluated outcomes at 12 and 10 weeks, respectively, we decided to assess the efficacy of filgotinib at eight weeks because of the rapid emergence of this drug's effectiveness. However, eight weeks must have been too short to evaluate the precise patient outcomes and safety profiles. Because this trial has an additional long-term trial of 44 weeks (for a total of 52 weeks), further evaluation, including on lymph nodes, is necessary. Despite these limitations, our findings demonstrate the filgotinib safety profiles of five enrolled patients over a limited duration.

## Conclusions

Based on the rationale that JAK contributes to the pathogenesis of iMCD, a phase Ib investigator-initiated trial of filgotinib in patients with iMCD for eight weeks has been conducted. The safety profile of filgotinib against iMCD is comparable to that against RA and UC over a short duration. The efficacy of filgotinib against iMCD was not evident after eight weeks. While the eight-week short-term use of filgotinib in iMCD appears to have an acceptable safety profile, further long-term evaluations of the safety profile and efficacy of filgotinib in the treatment of iMCD are essential.

## References

[1] Castleman B, Iverson L, Menendez VP (1956). Localized mediastinal lymphnode hyperplasia resembling thymoma. Cancer.

[2] Fajgenbaum DC, van Rhee F, Nabel CS (2014). HHV-8-negative, idiopathic multicentric Castleman disease: novel insights into biology, pathogenesis, and therapy. Blood.

[3] Mukherjee S, Martin R, Sande B, Paige JS, Fajgenbaum DC (2022). Epidemiology and treatment patterns of idiopathic multicentric Castleman disease in the era of IL-6-directed therapy. Blood Adv.

[4] Fujimoto S, Koga T, Kawakami A (2018). Tentative diagnostic criteria and disease severity classification for Castleman disease: a report of the research group on Castleman disease in Japan. Mod Rheumatol.

[5] Yoshizaki K, Matsuda T, Nishimoto N (1989). Pathogenic significance of interleukin-6 (IL-6/BSF-2) in Castleman's disease. Blood.

[6] van Rhee F, Stone K, Szmania S, Barlogie B, Singh Z (2010). Castleman disease in the 21st century: an update on diagnosis, assessment, and therapy. Clin Adv Hematol Oncol.

[7] Nishimoto N, Kanakura Y, Aozasa K (2005). Humanized anti-interleukin-6 receptor antibody treatment of multicentric Castleman disease. Blood.

[8] van Rhee F, Fayad L, Voorhees P (2010). Siltuximab, a novel anti-interleukin-6 monoclonal antibody, for Castleman's disease. J Clin Oncol.

[9] Van Rhee F, Wong RS, Munshi N (2014). Siltuximab for multicentric Castleman's disease: a randomised, double-blind, placebo-controlled trial. Lancet Oncol.

[10] van Rhee F, Voorhees P, Dispenzieri A (2018). International, evidence-based consensus treatment guidelines for idiopathic multicentric Castleman disease. Blood.

[11] O'Shea JJ, Schwartz DM, Villarino AV, Gadina M, McInnes IB, Laurence A (2015). The JAK-STAT pathway: impact on human disease and therapeutic intervention. Annu Rev Med.

[12] Villarino AV, Kanno Y, O'Shea JJ (2017). Mechanisms and consequences of Jak-STAT signaling in the immune system. Nat Immunol.

[13] Pierson SK, Shenoy S, Oromendia AB (2021). Discovery and validation of a novel subgroup and therapeutic target in idiopathic multicentric Castleman disease. Blood Adv.

[14] Genovese MC, Kalunian K, Gottenberg JE (2019). Effect of filgotinib vs placebo on clinical response in patients with moderate to severe rheumatoid arthritis refractory to disease-modifying antirheumatic drug therapy: the FINCH 2 randomized clinical trial. JAMA.

[15] Van Rompaey L, Galien R, van der Aar EM (2013). Preclinical characterization of GLPG0634, a selective inhibitor of JAK1, for the treatment of inflammatory diseases. J Immunol.

[16] Winthrop KL (2017). The emerging safety profile of JAK inhibitors in rheumatic disease. Nat Rev Rheumatol.

[17] Traves PG, Murray B, Campigotto F, Galien R, Meng A, Di Paolo JA (2021). JAK selectivity and the implications for clinical inhibition of pharmacodynamic cytokine signalling by filgotinib, upadacitinib, tofacitinib and baricitinib. Ann Rheum Dis.

[18] Liu AY, Nabel CS, Finkelman BS (2016). Idiopathic multicentric Castleman's disease: a systematic literature review. Lancet Haematol.

[19] Nishimura Y, Fajgenbaum DC, Pierson SK (2021). Validated international definition of the thrombocytopenia, anasarca, fever, reticulin fibrosis, renal insufficiency, and organomegaly clinical subtype (TAFRO) of idiopathic multicentric Castleman disease. Am J Hematol.

[20] Fukuhara S, Ware JE, Kosinski M, Wada S, Gandek B (1998). Psychometric and clinical tests of validity of the Japanese SF-36 Health Survey. J Clin Epidemiol.

[21] Feagan BG, Danese S, Loftus EVJ (2021). Filgotinib as induction and maintenance therapy for ulcerative colitis (SELECTION): a phase 2b/3 double-blind, randomised, placebo-controlled trial. Lancet.

[22] Källmark H, Bergström T, Nagel J, Gullstrand B, Einarsson JT, Bengtsson AA, Kapetanovic MC (2024). Serologic immunogenicity and safety of herpes zoster subunit vaccine in patients with rheumatoid arthritis receiving Janus kinase inhibitors. Rheumatology (Oxford).

[23] Verstovsek S, Kantarjian H, Mesa RA (2010). Safety and efficacy of INCB018424, a JAK1 and JAK2 inhibitor, in myelofibrosis. N Engl J Med.

[24] Lust H, Gong S, Remiker A, Rossoff J (2021). Idiopathic multicentric Castleman disease with TAFRO clinical subtype responsive to IL-6/JAK inhibition: a pediatric case series. Pediatr Blood Cancer.

[25] Kakutani T, Nunokawa T, Chinen N, Tamai Y (2022). Treatment-resistant idiopathic multicentric Castleman disease with thrombocytopenia, anasarca, fever, reticulin fibrosis, renal dysfunction, and organomegaly managed with Janus kinase inhibitors: a case report. Medicine (Baltimore).

[26] Gao YH, Duan MH, Li J, Zhang L (2024). Ruxolitinib for the treatment of refractory idiopathic multicentric Castleman disease: a case report. Turk J Haematol.

